# Enhancing E-Health Information Systems with Agent Technology

**DOI:** 10.1155/2009/279091

**Published:** 2008-12-01

**Authors:** Minh Tuan Nguyen, Patrik Fuhrer, Jacques Pasquier-Rocha

**Affiliations:** Department of Computer Science, University of Fribourg, 1700 Fribourg, Switzerland

## Abstract

Agent Technology is an emerging and promising research area in software technology, which increasingly contributes to the development of value-added information systems for large healthcare organizations. Through the MediMAS prototype, resulting from a case study conducted at a local Swiss hospital, this paper aims at presenting the advantages of reinforcing such a complex E-health man-machine information organization with software agents. The latter will work on behalf of human agents, taking care of routine tasks, and thus increasing the speed, the systematic, and ultimately the reliability of the information exchanges. We further claim that the modeling of the software agent layer can be methodically derived from the actual “classical” laboratory organization and practices, as well as seamlessly integrated with the existing information system.

## 1. Introduction

The business of today's complex organizations such as hospitals in a healthcare network relies on sophisticated information systems which often inherit many weaknesses from the past. For instance, due to its lack of flexibility, a legacy information system cannot integrate the ever-increasing requirements in order to assist the users or to free them from many routine tasks. (A legacy information system represents a massive, long-term investment in the past [1], with poor system quality, design, and architecture. It is costly to adapt to rapidly changing business requirements.) This weakness of legacy information systems is one of many aspects of the “automation gap.” Another major weakness relates to the increasing physical mobility of users. Many legacy information systems are designed for users working at fixed client workstations in fixed offices. They do not take into account recent advances in mobile technology such as PDAs, mobile phones, and smartphones. In many legacy information systems, the information flow still requires human interaction between actors either face-to-face or through the plain old telephone communication system to get things done (information delivery, alert sending, people search, feedback, etc.). Automation gap, lack of mobility, and direct human interaction result in an inefficient information flow and data processing:

- nonautomated information search and retrieval are time-consuming;
- errors may occur in data transmission by humans;
- users must be physically present at either end of the communication link to successfully establish a conversation (i.e., only synchronous interaction);
- the lack of a systematic activity log makes it difficult to determine the responsibilities of actors when problems or errors occur during a business process.

This research aims at applying a systematic agent technology approach to overcome these weaknesses. The design of a software agent layer on top of a legacy information system offers many advantages to users:

- it adds interesting properties to the information system: ubiquitousness, intelligence, scalability, systematic management, logging of the information flows, and so forth;
- it helps humans to interact efficiently among themselves and with the information system. Indeed, human effort and time can be saved by transferring routine tasks from humans to software.

After this first introductory part, Section 2 provides background information on software agents, agents platforms, and development methodologies in general.

Section 3 presents a case study conducted at the HCF Laboratory (HCF is the French acronym for Hospital of the state of Fibourg, Switzerland) . This section is further divided as follows:

- the mission and the information system of the HCF Laboratory are presented;
- the weaknesses and potential problems of the current information system are identified;
- finally, a software agent-based solution to enhance the system is proposed.

Section 4 focuses on the medical multiagent system (MediMAS) prototype, which represents our first implementation of the proposed agent-based solution. It simulates an end user's (lab personnel, physician) point of view by considering software agents as personal assistants and by showing them in action.

Section 5 shows how it was possible to define the requirements and to sketch the architecture of the prototype using a well-defined and systematic approach, and this section also briefly describes its main components.

Finally, Section 6 concludes this paper by summarizing the main achievements of our work and by discussing some extensions and improvements planned for the future.

## 2. Background

It is out of the scope of this paper to offer full background information on software agents and their related technologies. Therefore, the three next subsections only provide a short introduction to the domain and refer the interested reader to the abundant literature for further details.

### 2.1. What is an Agent?

The term “agent” appears in a wide spectrum of research areas such as economics, physics, biology, mathematics, artificial intelligence, and software engineering. Therefore, a unified notion of agent is difficult to extract from the research literature. In this section, we do not aim to coin a new definition, but to highlight the fundamental properties of agents from two published definitions.

**Definition 1**

An autonomous agent is a system situated within and a part of an environment that senses that environment and acts on it, over time, in pursuit of its own agenda and so as to effect what it senses in the future [2].

**Definition 2**

An agent is a small, autonomous, or semiautonomous software program that performs a set of specialized functions to meet a specific set of goals, and then provides its results to a customer (e.g., human end-user, another program) in a format readily acceptable by that customer [3].

The first definition proposes the most general notion of agent which may be a person, a robot, a piece of software, and so forth. The second definition focuses on agents in the software domain which is of interest to us. Both definitions exhibit the following basic properties of software agents:

- autonomy: agents have some degree of control over their actions and can work without intervention of humans;
- social ability: agents can coordinate their actions and cooperate with other agents to achieve their goals, using a common language to communicate with each other;
- reactivity: agents can perceive their environment and respond to environmental changes;
- proactiveness: agents can act on their own initiative to achieve their goals instead of simply reacting with the environment.

For our research purposes, we further characterize a software agent as *a running program object, capable to initiate, receive, execute, or reject a message autonomously to attain its goals during its life cycle*.

### 2.2. Agent Platforms

An agent platform is a software environment in which agents are incarnated and operate to achieve their goals. The agent platform must provide the following minimum set of functionalities [4, 5]:

- agent management (creating, starting, removing, migrating agents, etc.),
- agent communication,
- supervision of agents, error notification,
- security mechanism.

Today, several platforms have been developed (e.g., JADE [6], JACK [7], AgentBuilder [8], Aglet [9], etc.) and researches are being conducted to define new platforms for building agent systems. JADE was selected based on two criteria:

- the selected platform is well-proven;
- it is scalable for our research and experimental purposes.

Java Agent DEvelopment Framework (JADE) is a software framework fully implemented in the Java language. It simplifies the implementation of multiagent systems through a middleware that complies with the Foundation For Intelligent Physical Agents (FIPA) specifications and through a set of graphical tools that supports the debugging and deployment phases. (FIPA is an IEEE computer society standards organization that promotes agent-based technology and the interoperability of its standards with other technologies [10].) This agent platform can be distributed across machines (which do not even need to share the same OS) and the configuration can be controlled via a remote GUI. JADE has been developed by the Telecom Italia Lab [6], and the Agent and Object Technology Lab at the University of Parma [11]. It is open-source, cost-free, and offers the developer complete control over the framework. We refer the interested reader to [12] for a good introduction to the JADE agent platform.

### 2.3. Agent-Oriented Methodologies

The concept of agents was first introduced in the 1970's. However, the development of agent-based systems is a relatively new domain of software engineering. Today, several agent-oriented methodologies have been developed (e.g., Gaia [13], MaSE [14], and MAS-CommonKADS [15]). They are based on different theoretical foundations [16]: artificial intelligence (AI), object-oriented Programming (OOP), combination of AI and OO, as well as i* organization modeling framework (Tropos) [17].

These methodologies contribute significantly to the rigorous and systematic development of agent-based systems. The JADE_Methodology [18] is a new agent-oriented methodology that supports the ontology approach. It encompasses the analysis and design phases to develop software agents on the JADE platform. This methodology proposes to build the ontology at the end of the design phase in order to share the knowledge between software agents.

## 3. HCF Laboratory: Current Organization and Software Agent Solution

### 3.1. The HCF Laboratory

The HCF Laboratory [19] provides medical analysis ordered by hospitals in the state. The laboratory is located on several sites with different domains: haematology, immuno-haematology, chemistry, and microbiology. It receives daily hundreds of orders with specimens, analyzes the specimens, then delivers final results to the requesters (doctors, hospital departments, etc.). The method of transmission of test results depends on their urgency level.

Besides the lab equipment for carrying out medical analysis, the personnel of the HCF Laboratory are supported in their daily tasks by the WinDMLAB Multisite laboratory information system [20], coupled with a traditional telephone communication system. They constitute two major components of the current HCF Laboratory Information System (cLIS).

cLIS ensures the availability of medical results in a centralized database and their transmission:

- between departments and sites of the laboratory,
- between the laboratory and the HCF,
- between the laboratory and other requesters in the province of Fribourg.

Each requester (doctors, hospital departments, etc.) can access and review the test reports on their patients at any level of detail.

The WinDMLAB Multisite system and the traditional telephone communication system must coexist to achieve all the functionalities as cLIS was initially designed for. Indeed, several scenarios still require the telephone communication system to get things done, for example, in the following circumstances:

- a lab technologist calls a physician to transmit patient's test results;
- a physician calls the laboratory to obtain by phone the test results;
- a lab technologist asks, by phone, his director to make a decision in an emergency situation, and so forth.

**Figure 1:**
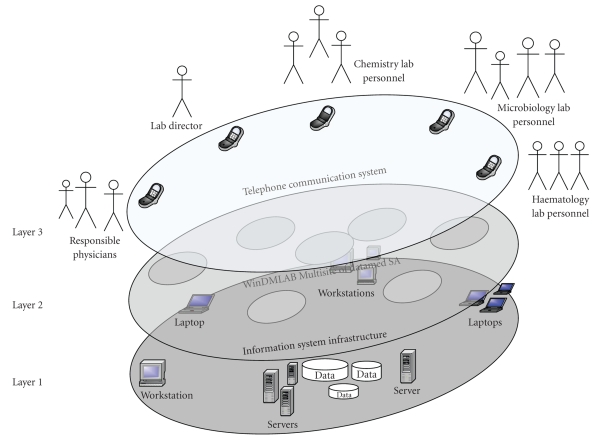
Layers of the current laboratory information system.

Figure 1 illustrates cLIS as a three-layer system in which both the laboratory information system and the telephone communication system coexist:

- the first layer defines the information system infrastructure, which is composed of servers running different operating systems and application software in a computer network;
- the second layer is the WinDMLAB Multisite system;
- the third layer provides the telephone communication system which allows requesters and laboratory staff to exchange test results via voice and fax.

One can notice that human actors interact with each other directly or indirectly through the second and third layers.

### 3.2. Potential Problems

cLIS raises numerous potential problems [21]:

- even though the major part of results (80%) are transferred through automats and WinDMLAB Multisite system, the quality of services provided by cLIS depends to a more or less extent on human factors, for example, any mistake of a lab technologist in transferring test results to a doctor may cause dramatic consequences on patients;
- cLIS does not allow the requesters to know when results become available;
- the processes which take place in the telephone communication system (layer 3) cannot be logged automatically in cLIS for monitoring and tracking purposes;
- physicians who use cLIS spend a lot of time searching, retrieving, consulting, and interchanging the test results;
- to establish a successful phone communication, two actors must be present, therefore, time is wasted if either one cannot reach the other when needed;
- because of the time-consuming use of cLIS in many scenarios, physicians and laboratory personnel have less time for their real medical activities.

The above-identified problems, caused by human operations, often prevent information to flow smoothly from cLIS to actors and vice versa. These problems illustrate the so-called “automation gap” [22, 23]. What is needed is a systematic, strategic approach that automates error-prone human processes.

### 3.3. A Software Agent Solution

The “automation gap” may be filled using different software technologies, for example, JavaSpaces with SMS message technology, Web services technology, multiagent technology, and so forth. It is out of the scope of this paper to compare these technologies. Our purpose is to propose a methodology for allowing us to migrate from the legacy human agent-centered cLIS toward an enhanced software agent-based system. In cLIS, actors (laboratory personnel, laboratory director, physicians, etc.) are human agents. A human agent is a professional characterized by experience, skills, intelligence, reactiveness, proactiveness, and ability to work autonomously and to cooperate with other human agents. They also have weaknesses inherent to human beings. Our proposal aims at designing software agents which will work on behalf of human agents with similar characteristics. In other words, our solution delegates daily routine tasks performed by human agents to software agents. In this new approach, each actor is assigned a personalized software agent which acts as his personal assistant. We also say that the actor is an assistant's owner. When talking about these personal assistants, we could also use the “virtual twin” metaphor [24] or consider them as avatars representing humans like in virtual worlds. The assistant receives a list of things to do from its owner, performs the assigned tasks in close cooperation with other software agents, and delivers the final result to the owner. In our solution, the software agents are designed on Layer 3, shifting the telephone communication system up to the fourth layer (cf. Figure 1). The software agent solution offers significant advantages for cLIS:

- the features and functionalities of WinDMLAB Multisite are maintained, preserving the investment in this legacy laboratory application;
- in the new software agent-based cLIS, the delegation of routine tasks from human to software agents (personal assistants) allows human actors to focus their attention on specimen analysis, test result interpretation, medical decision making, and so forth;
- the new software agent-based cLIS, coupled with mobile devices (PDAs, mobile phones, smartphones, etc.), allows the actors to view the test results transmitted by personal assistants anywhere and anytime;
- all events and actions are systematically logged and centralized to support auditing of the system. Traceability and exception investigation, for example, to answer a patient's complaint, is also improved.

## 4. The MediMAS Prototype

The MediMAS prototype [21] is the first experimental implementation of the proposed agent-based solution. A case study was conducted at the HCF Laboratory to test it in the real world, and to explore different practical aspects.

### 4.1. Agents as Personal Assistants

MediMAS has six agent categories:

- physician agents,
- lab personnel agents,
- lab director agents,
- alert manager agent,
- integration agent, and
- audit agent.

**Figure 2:**
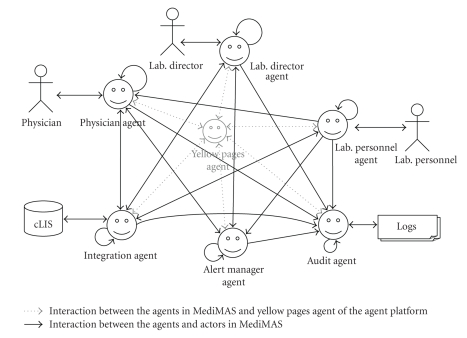
MediMAS overview.

Figure 2 depicts their organization in which the agents assist different categories of humans in their daily tasks. This figure also shows the social ability of agents to cooperate with each other in order to automate the information flow between the actors themselves, as well as between the actors and the cLIS.

### 4.2. Software Agents in Action

#### 4.2.1. Environment Setup

In the environment of our MediMAS prototype, the integration agent (riAgent) plays a central role. Therefore, it is launched first with the JADE platform before starting any other agent. When the setup is complete, the agents are attached to the MediMAS's containers (a JADE container is a runtime environment for agents [25]):

- riAgent is the integration agent,
- amAgent is the alert manager agent,
- adAgent is the audit agent,
- pAgents are the physician agents,
- lpAgents are the lab personnel agents,
- ldAgents are the lab director agents.

In the MediMAS system, each human actor (physician, lab personnel, lab director) is assigned an Agent, and simultaneously, one or more GuiAgents. For example, a single agent pAgent TuanAgent and two GuiAgents are assigned to the physician Tuan.

We now setup our sample WinDMLAB database by feeding it with the fictitious test results of specimens nlab-007, nlab-008, and nlab-009 in order to simulate the three test results which are recorded into the database by the lab analysers, and validated by the lab technologist. (Our sample WinDMLAB database was developed using SQLite RDBMS [26].)

Let us introduce the actors who will play different roles in our scenario:

- Tuan is a physician in the HCF and is assigned the ID 3;
- Jacques is the lab director;
- Patrik is a lab technologist in HCF Laboratory: he is working on the specimens: nlab-007, nlab-008, and nlab-009, ordered by a caregiver Tuan.

In the following scenario, starting with the notification of results availability, we study in finer detail the human actors, their assigned personal assistant agents, and their interactions.

#### 4.2.2. Notification of Results Availability

**Figure 3:**
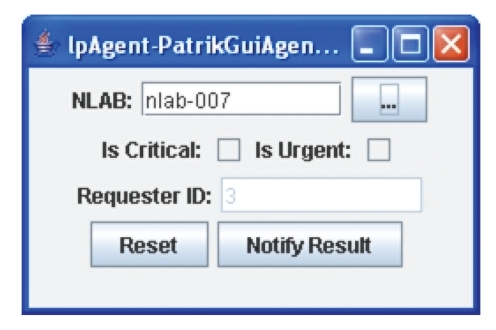
Patrik's laboratory personnel agent GUI.

- Patrik has finished the analysis of all specimens. The three test results are recorded into WinDMLAB database. Table 1 shows the priority of the specimens and their degree of criticality. (The priority of an analysis is set by its requester and the degree of criticality depends on its result and is set by the lab technologist.)
- At completion of the nlab-007 analysis, Patrik observes that the test results are noncritical (see Table 1). In order to notify the availability of the test results to Tuan (requester ID = 3), Patrik enters nlab-007 and clicks on the button beside the NLAB field to automatically fill in the other fields (Figure 3). Finally, Patrik clicks the “notify result” action button to direct his lpAgent to announce the availability of test results to the requester.
- Patrik further treats the other results in the same manner.
- Patrik's lpAgent sends the announcements of the results to Tuan's pAgent.
- It also sends these announcements to amAgent which records the announcements and starts to monitor closely the read/unread status of the new test results.

#### 4.2.3. Acknowledgments of Notification Receipt

**Figure 4:**
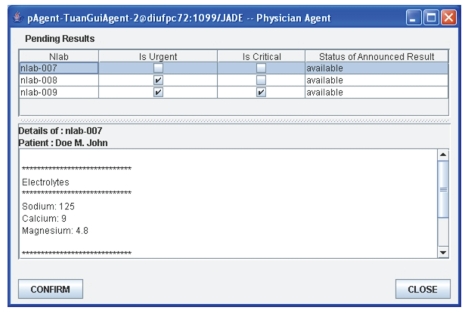
Tuan's physician agent GUI.

**Figure 5:**
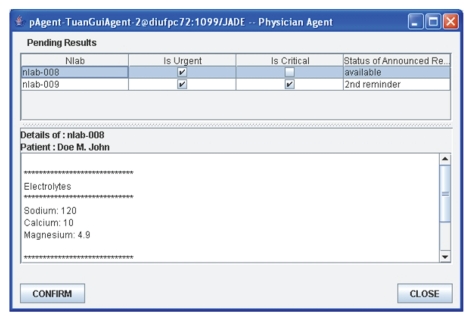
Tuan's physician agent GUI—after nlab-007 has been confirmed.

- Concurrently with amAgent, Tuan's pAgent receives the announcements and refreshes the list of pending results in the upper pane of its window by adding the new announcements of nlab-007, nlab-008, and nlab-009 test results, flagged as “available” in the status of announced Result column (Figure 4).
- Tuan clicks on the received announcement nlab-007 in the list of pending results in order to preview the details of the test results. Tuan's pAgent requests riAgent to retrieve the contents of the nlab-007 test results and displays the contents of the nlab-007 test results in the lower pane of its window (Figure 4).
- Tuan clicks the “confirm” button to acknowledge receipt of the notified announcement of nlab-007 and thus directs his pAgent to send this acknowledgement to amAgent.
- amAgent updates the status of nlab-007 as “read” and removes the nlab-007 announcement from his own internal list. This terminates the monitoring of nlab-007 by amAgent.
- Once the announcement is flagged as “read,” Tuan's pAgent removes nlab-007 from the list of pending results (Figure 5).
- Tuan further acknowledges the nlab-008 result.

One notices that, in the pAgent's window, each announcement is first flagged as “available” during a predefined time interval, for example, 20 minutes for normal test results; and 10 minutes for critical ones. Thanks to the close monitoring of pending announcements, amAgent alerts pAgent as soon as an announcement has not been confirmed within the predefined time interval. pAgent immediately flags the alerted announcement as “1st reminder,” then “2nd reminder,” and so on in the Status of Announced Result column.

#### 4.2.4. Problem Detection and Alert

**Figure 6:**
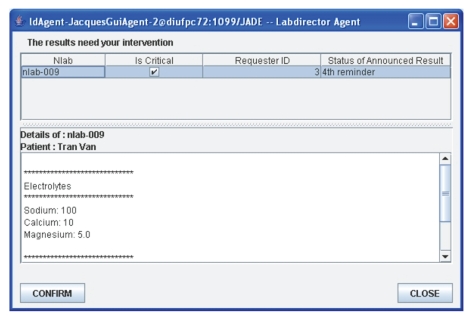
Jacques' lab director agent GUI.

- For the nlab-009, amAgent has not yet received an acknowledgment message from Tuan's pAgent within the preset time interval. After three unsuccessful warnings, amAgent escalates up the organizational hierarchy by sending an alert to Jacques' ldAgent.
- Jacques' ldAgent receives the nlab-009 alert from amAgent and displays it in the ldAgent's window (Figure 6).
- Jacques clicks on the nlab-009 alert in order to preview it. Jacques's ldAgent requests riAgent to retrieve the contents of the nlab-009 test result and displays the contents of the nlab-009 test results in the lower pane of its window.
- Jacques contacts Tuan to manually transmit the test results to him.
- Jacques clicks the “confirm” button to acknowledge receipt of the nlab-009 alert and thus directs his ldAgent to send this acknowledgment to amAgent.
- amAgent updates the status of nlab-009 as “read,” and removes the nlab-009 announcement from his own internal list. This terminates the monitoring of nlab-009 by amAgent.
- Once the announcement is flagged as “read,” Jacques's ldAgent and Tuan's pAgent remove nlab-009 from their respective windows.
- Throughout the above-simulated scenario, each agent sends to the audit agent (adAgent) the start and stop times of every performed task along with its relevant information (date and time, involved actors, action, etc.).

We have simulated some specimens to demonstrate the working of assistant agents in the MediMAS prototype and the benefits of a software agent approach to enhance a legacy information system. In order to fully grasp the power of our solution, one however must consider the real laboratory, where hundred of specimen analysis are ordered everyday by dozen of physicians. After a rather simple configuration process, each human actor will be able to transparently rely on his software counterpart to be reminded what he has to do next with respect to the hospital regulations. Furthermore, all communication exchanges and reminder warnings will be coordinated, timely delivered to all the appropriate actors, and properly logged for further references.

At this stage, the attentive reader has certainly noticed that we used a very high level approach in order to describe the concrete run-time working of the MediMAS prototype. It is, however, very important for her to understand that MediMAS components are not just plain objects, but they are, indeed, software agents in the sense of the definition given at the end of Section 2.1. Because of that, the use of agent technologies in general and of an agent platform in particular is a necessity if one does not want to reinvent the wheel by implementing from scratch many low-level services such as naming and yellow pages services, code mobility support, debugging and monitoring/management facilities, security mechanism, agent communication, or resource control. For example, the alert manager agent, amAgent introduced above, is a running program object, with its own thread of control (i.e., having its own autonomy), which

- reacts to physician and lab personnel agents messages by updating its test results pending list;
- has an aim to timely detect and to act upon test results with abnormal unread status;
- acts autonomously (i.e., without the necessity of a special external event or method call) in order to fulfill its goal. It does so by constantly monitoring its test results pending list and by sending warning messages to the appropriate agents (physician and lab director ones) according to the hospital regulations.

Messages are based on the FIPA ACL Message standard [10], and the behaviors or agent “intelligence” are programmed in Java classes using either plain procedural code or declarative rules with the help of the Jess to JADE Toolkit developed by our research group [27]. Note that with the latter technology, it is even possible to change the agent behavior by modifying rules at run-time (e.g., escalating up the organizational hierarchy after two instead of three unsuccessful warnings or warning another physician in the same group if available instead of the lab director).

## 5. Development Methodology

**Figure 7:**
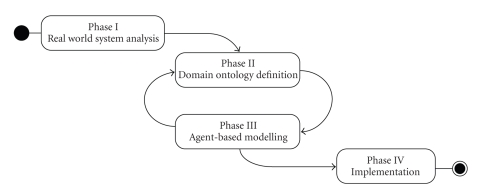
The phases of the methodology.

**Figure 8:**
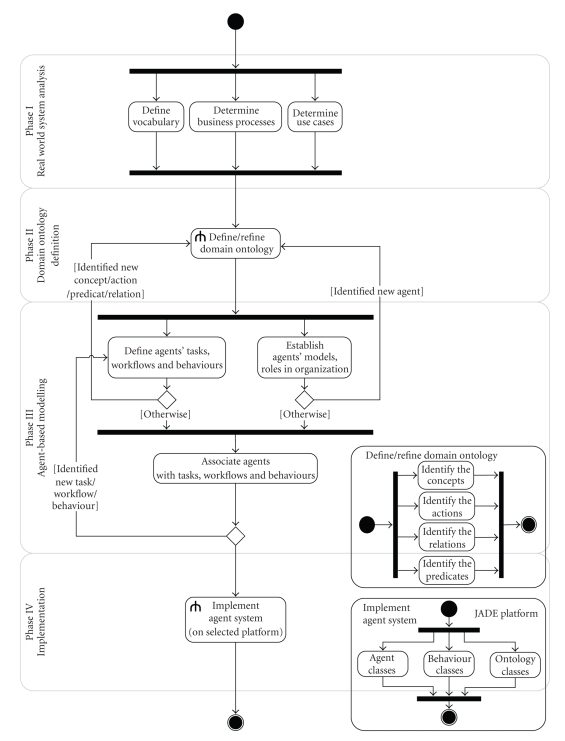
Development methodology.

We have designed our own “in-house” methodology, inspired by the theoretical foundations mentioned in Section 2.3. More precisely, we adapted the JADE_ Methodology [18] to our own purposes by integrating the ontology in the earlier phases of the modelling process. Our strategy has been applied to develop the MediMAS prototype. The next paragraphs present it in four phases (see Figure 7), while Figure 8 summarizes it and put in evidence the relationships between its different phases.

### 5.1. Phase I: Real-World System Analysis

**Figure 9:**
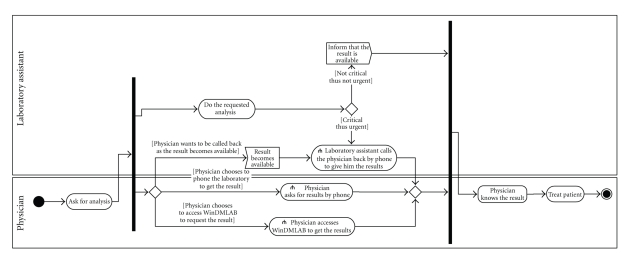
The business processes of the HCF laboratory.

The analyst perceives the current system in order to understand its goals, problems, and its future requirements. This phase aims at defining a common vocabulary and describing the current organization of entities (actors, human agents), use cases, and/or business processes of the system. The deliverables of Phase I consist in a well-defined set of goals and requirements, the common vocabulary describing the entities with their organization, a set of identified use cases, and business processes. In our case study, the outputs of our real-world system analysis are the three-layer information system structure of the HCF Laboratory (Figure 1), and UML activity diagrams of its business processes (Figure 9). 

### 5.2. Phase II: Domain Ontology Definition

The Domain Ontology Definition phase takes the deliverables of Phase I as input and aims at defining the domain or application terminology standards and semantics. To this end, the analyst focuses on concepts, actions, predicates and relations between concepts. In MediMAS, we adopt the following guidelines to build the ontology:

- Concepts are substantives (e.g., doctor, patient, analysis, etc.).
- Actions are verbs or verbal phrases (e.g., SendResult, Alert, SendAvailableList, etc.).
- Predicates are expressions that make statements about something, which can be evaluated true, false or indeterminate (e.g., isTestResultCritical, isResultComfirmed, etc.).
- Relations are expressions that establish the relationship between concepts.

The output of this phase is the domain or application ontology, that actors will use to understand each other in their communications.

In software engineering, ontology development tools, such as Protégé [28], TopBraidComposer [29], etc., have been developed in order to assist the ontologists to build the domain or application ontology efficiently. The interested reader is refered to [30] for a graphical overview of the ontology we defined using the Protégé suite of tools.

### 5.3. Phase III: Agent-Based Modelling

The modelling phase consists in the following set of tasks using the deliverables of Phase I and II as inputs:

- identify and create eligible software agents which will be assigned to actors;
- determine the tasks (also called the responsibilities) of each agent;
- specify the workflow of elementary operations in each task and the agent's operational behavior;
- assign tasks, workflows, and behaviors to agents according to their roles in the organization.

Figures 7 and 8 draw our attention to the iterative nature of the tasks within Phase III on one side, and between Phases II and III on the other side. Indeed, successive refinement steps are required in order to enrich the domain ontology as new concepts, actions, predicates, and relations between concepts are identified.

The deliverables of this phase are the documents:

- describing the agents in different categories, and
- specifying all the tasks, workflows, and behaviours, and their assignment to agents.

The agent categories and their assigned tasks in MediMAS are summarized in Table 2.

### 5.4. Phase IV: Implementation

The previous phases are platform-independent. In Phase IV, the selection of a platform closely impacts the implementation process. In our case study, the JADE platform was selected to implement the MediMAS prototype.

This phase involves the programmer team to implement and test the agent-based system according to the model specifications. To this end, the programmers use the deliverables of the previous phases as inputs, and then translate them into system components which are extensions of the existing classes in JADE, namely:

- designed agents are translated into classes of agents according to the terminology used in JADE;
- designed tasks, workflows, and behaviours are converted into classes of behaviours in the sense of JADE.

The domain ontology must also be implemented as extensions of the existing ontology in JADE. This task is achieved:

- either by manually coding vocabulary, bean classes, ConceptSchema, AgentActionSchema, Predicate-Schema, and so forth, or
- through the bean generator plug-in for Protégé [31].

The completion of phase IV results in a multiagent system that fulfils the defined user goals and requirements and operates on the selected platform. It would be out of the scope of this article to fully describe the software architecture of the MediMAS prototype. It is nevertheless worth giving an overview of its main software components. (The interested reader can find the class diagram of MediMAS as implemented on the JADE platform in [30] and its complete source code is available at [32].)

**Figure 10:**
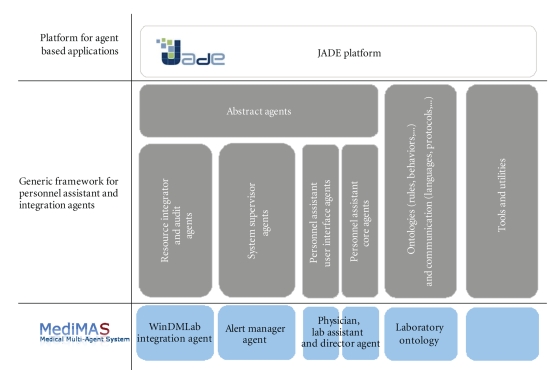
Overview of the software architecture.

A typical layered approach has been adopted (see Figure 10): the upper layer is an abstract layer providing the basic classes, interfaces, and agent types, and it directly extends the JADE platform. The second layer offers the main functionalities and default behaviors for each kind of identified agent type: resource integration for seamless interfacing with legacy systems, audit agent for addressing logging issues, and alike system supervisor agents which enhance the system with some new services and the personnel assistant agents which embody the “virtual twin” paradigm. Note that this latter category is split into core and user interface agents. This separation allows for a one-to-many relationship between a personal agent's core part and several user interface agents which are deployed on the humans' computing devices (desktops/laptops and/or smartphones and/or web browsers, etc.).

These agent families form the main vertical blocks of our architecture. Eventually, the lowest layer is dedicated for application specific implementations of the agents. In the case of MediMAS, this layer contains

- the WinDMLAB integration agent,
- the alert manager agent,
- the lab assistant, lab director and physician personnel assistant agent.

Beside these blocks, there are two further components (rightmost on Figure 10): one for ontology related issues and one for miscellaneous tools and utilities.

This layered architecture actually provides a general framework that could be used for other application domains than our medical laboratory use case. In order to reuse the framework, one could simply inject a new ontology, attach the according behaviours to the personnel assistant agents, and implement the business logic of the system supervisor agents.

## 6. Conclusion

This research paper discusses major features and benefits of our agent-based approach to enhance a hospital laboratory legacy information system. Such approach preserves the investment in the legacy system and allows developers to seamlessly add new features, which aim at filling the automation gap, satisfying the needs of growing user mobility, and providing intelligent assistance to users. Finally, a methodology to systematically adopt and implement such a solution is proposed and it is validated with the implementation of the concrete MediMAS prototype.

### 6.1. Achievements

The current version of the MediMAS prototype provides physicians, lab personnel, and lab director with software agents running on desktop computers. (The whole source code and related documentation are available for download from [32].) These agents act as personal assistants to free the actors from tedious and routine work so that they can really concentrate on their medical activities.

### 6.2. Work in Progress

#### 6.2.1. Mobile MediMAS

Our research will extend the model to allow software agents to run on mobile devices (e.g., PDAs, mobile phones, smartphones, etc.). The agents that work for the same owner on different devices must collaborate and synchronize their tasks to efficiently assist the owner who may work anywhere and anytime. A first prototypal version of this extended model is already available [32, 33], but still needs some fine tuning.

#### 6.2.2. MediMAS Simulation Tool

The development of a simulation tool for MediMAS is another topic of our research. The tool offers the HealthCare experts the opportunity to visualize the working of MediMAS prototype by simulation, and to get an insight in the properties of an agent-based system in the HealthCare domain (ubiquitousness, intelligence, reactiveness, proactiveness, scalability, etc.). A first version of the tool is now available [34] and has been extensively used in order to debug and test the MediMAS prototype.

#### 6.2.3. Adaptive MediMAS Agents

Withing another project, we developed the Jess to JADE (J2J) toolkit [27], which allows JADE agents to seamlessly use the Jess rule engine [35] in order to perform appropriate behavior. This solution has been tested on our alert manager agent and it allowed us to declaratively define and modify the agent behavior at runtime.

#### 6.2.4. Methodology Enhancement

The light in-house agent-based system design methodology has been defined, and applied in the MediMAS experimental project in HealthCare domain. Future extensions will enhance the methodology with additional modelling possibilities to design more complex real-world systems.

## Figures and Tables

**Table 1:** The three simulated specimens.

Criticality/priority	None	Urgent
Non-critical	nlab-007	nlab-008
Critical	—	nlab-009

**Table 2:** Tasks performed by agent categories.

Agent categories	Tasks
Physician agent	Receives notification of test results availability from the lab personnel agents.
	Receives alerts of unread available test results from the alert manager agent.
	Notifies the physician that test results are available.
	Queries the integration agent for test results according to search criteria determined by the physician.
	Receives test results data from the integration agent.
	Displays test results data to the physician.
	Informs the alert manager agent about the read/unread status of the test results sent to the physician.
	Informs the audit agent before and after each action.

Lab personnel agent	Notifies the alert manager agent that test results are available.
	Notifies the physician agents that results are available.
	Informs the audit agent before and after each action.

Lab director agent	Receives alerts from the alert manager agent signaling the abnormal unread status of a test result.
	Reports alert to the lab director.
	Acknowledges the alert manager agent that the lab director read the alert sent to him.
	Informs the audit agent before and after each action.

Alert manager agent	Alerts the lab director agent as soon and as the abnormal unread status of a given test result is detected.
	Receives test results from the lab personnel agent.
	Receives from the physician agent the status “test results have been read by physician.”
	Receives from the lab director agent the status “alert message has been acknowledged by the lab director.”
	Informs the audit agent before and after every action.

Integration agent	Retrieves test results from cLIS, based on the query issued by the physician agent or the lab director agent.
	Delivers extracted test results to the requester agent.
	Informs the audit agent before and after every action.

Audit agent	Receives the actual action start/end notifications and log them with their date and time.
